# Wearable sensor shown to specifically quantify pruritic behaviors in dogs

**DOI:** 10.1186/s12917-018-1428-x

**Published:** 2018-04-03

**Authors:** Joel D. Griffies, Jason Zutty, Marcel Sarzen, Stuart Soorholtz

**Affiliations:** 1 Animal Dermatology Clinic, Marietta, GA USA; 2 Georgia Institute of Technology, Atlanta, GA USA; 3 AgLogica Holdings, Inc, Norcross, GA USA

**Keywords:** Wearable sensor, Behavior monitor, Canine pruritus, Scratching, Head shaking, Dermatology, Accelerometer

## Abstract

**Background:**

Wearable technology is an exciting new field in humans and animals. In dogs activity monitors have helped to provide objective measurement tools where pet owner observation had been the only source of information. Previous research has focused on measuring overall activity versus rest. This has been relatively useful in determining changes in activity in orthopedic disease or post-surgical cases [Malek et al., BMC Vet Res 8:185, 2012, Yashari et al., BMC Vet Res 11:146, 2015]. Assessment of pruritus via changes in activity, however, requires an assumption that increased activity is due to scratching or other pruritic behaviors. This is an inaccurate method with obvious flaws as other behaviors may also register as greater activity. The objective of this study was to validate the ability of a multidimensional high frequency sensor and advanced computer analysis system, (Vetrax®, AgLogica Holdings, Inc., Norcross, GA, USA) to specifically identify pruritic behaviors (scratching and head shaking). To establish differences between behaviors, sensor and time stamped video data were collected from 361 normal and pruritic dogs. Video annotations were made by two observers independently, while blinded to sensor data, and then evaluated for agreement. Annotations that agreed between the two were used for further analysis. The annotations specified behaviors at specific times in order to compare with sensor data. A computer algorithm was developed to interpret and differentiate between these behaviors. Test subject data was then utilized to test and score the system’s ability to accurately predict behaviors.

**Results:**

Results for prediction of head shaking behavior included sensitivity and specificity of 72.16% and 99.78% respectively. Analysis of scratching produced sensitivity and specificity of 76.85% and 99.73% respectively. These results illustrate the ability of the system to accurately report both scratching and head shaking with an overall accuracy of 99.24% and 99.56% respectively.

**Conclusions:**

This study validates the use of this system to accurately and objectively report scratching and head shaking in dogs. While a small portion of scratching or head shaking behaviors may be missed, as indicated by the sensitivity, when detected, the confidence that these behaviors occurred is extremely high. These factors make this system a very useful tool for objective assessment of pruritus in clinical and research settings.

## Background

Pruritus is a common presenting complaint for dog owners and often a primary determinant of successful management in cases of skin and ear disease. It can be manifested in a variety of ways including scratching, rubbing, paw licking, head shaking, and others. These behaviors may vary both in distribution and intensity depending on the cause and degree of pruritus and the degree to which it is expressed in individual pets. Measurement of pruritus in dogs is challenging because it relies on the observations of pet owners. Obvious flaws in owner assessment of pruritus include the amount of time an owner observes the animal each day and the variability of an individual’s interpretation of the severity of pruritus. When presented to a veterinary practice, communication of the level of pruritus may be further influenced by the veterinarian or the veterinary clinic environment itself. Previous efforts to provide more consistent and reliable assessment of pruritus have included both visual analog scales and numerical scales that are presented to owners. Visual analog scales have been an improvement over standard numerical scales, but interobserver repeatability is still problematic as it continues to rely on subjective observations of the pet owner. Assessment of visual analog scales and numerical scales using video of pruritic dogs shown to first year veterinary students demonstrated poor consistency and repeatability among observers when using these methods. This was especially prevalent with mid-range to lower levels of pruritus [1]. A more refined pruritus visual analog scale (PVAS) was developed by Hill et al. with more specific behavior descriptions to guide an owner’s rating [2].

Limited research has been conducted using wearable sensors in an attempt to measure canine pruritus. Studies reported to date utilized differences in overall activity between atopic and normal dogs as measured by differences in total piezo-electric voltage generated during specific periods of time [3–5]. Results often indicated significantly greater activity for atopic dogs over healthy dogs, with increased activity particularly marked overnight. Of the weaknesses cited was the assumption that increased activity in atopic dogs was due to pruritus without evidence that this was true. Additional studies verified the association between increased nocturnal activity and nocturnal pruritus by videotaping kenneled dogs while measuring activity in the same manner [5]. While significant, exact pruritic behaviors could not be specified. Authors of another study observed that activity monitor data and observed behavior did not always coincide indicating a lack of specificity [4]. Each of these studies suggested increased overall activity in pruritic atopic dogs and sleep disturbance characterized by the increased activity during night-time hours. Data collection in these studies relied on changes in electric voltage generated over either 15 s or 1 min blocks of time. Activity during these times generated electric voltage regardless of the type and was dependent only on movement of the sensor in any direction. Each of these studies also summarized activity during specified time periods. This summary was intended to provide an overall representation of activity and was chosen at times of day when activities such as exercise were least expected (e g. night time hours). Three-dimensional movement detection and continuous sampling were not possible with this system. One previously-reported study attempted to classify specific behaviors using a multidimensional sensor [6]. This study evaluated the ability of a collar-based sensor to correctly identify behaviors in a small population of dogs (*n* = 13). Specific focus on pruritic behaviors was not attempted and scratching was not one of the behaviors researched. In this study a confusion matrix of individual behaviors was presented but accuracy for behaviors was summarized in a proof of concept fashion with the accuracy and testing of individual behavior analysis not reported. Based on previous research, a clear objective evaluation system to quantify pruritus in dogs is currently lacking that would support veterinary professionals with information adequate to help with decisions on treatments needed or success of those prescribed.

The objective of this study was to validate the ability of a multidimensional high frequency sensor and an advanced computer analysis system, to specifically identify pruritic behaviors (scratching and head shaking).

## Methods

### Overview

To establish a model of data collection and interpretation capable of identifying specific behaviors, two primary components were utilized: a multidimensional high frequency sensor and a computerized analytics model developed to interpret data and identify specific behaviors.

### Data collection

To collect examples of behaviors, dogs were observed and video recorded at 2 humane society facilities (HS1, HS2) and a dermatology referral practice (DR1). Dogs were chosen at HS1 and HS2 based on availability, good health as reported by caretaker staff and personality that allowed handling, placement of the collar, observation and video recording. Dogs were chosen at DR1 based on owner report of pruritus of any kind. Information on each subject dog was documented including name and weight. Breeds were also recorded when known (DR1) or were estimated (HS1, HS2; Table 1).

**Table 1:** Dogs utilized by Breed

	Location	
Breeds	HS1, HS2	DR1
Akita Mix	1	
American Bulldog		4
American Hairless		1
Australian Cattle Dog	6	
Basenji Mix	1	
Basset Hound		1
Beagle		1
Beagle Mix	9	
Bichon		2
Border Terrier		2
Boxer		2
Boxer Mix	13	2
Bull Terrier		1
Cav King Charles Spaniel		2
Chihuahua Mix	5	
Chow Mix	1	1
Cocker Spaniel		2
Cock-A-Poo		1
Collie Mix	10	
Coonhound Mix	8	
Corgi Mix	4	
Dachshund		2
Dachshund Mix	2	
Doberman Mix	2	
English Bulldog		1
English Bulldog Mix	12	
English Springer Spaniel		1
Fox Terrier		1
French Bulldog		4
German Shepherd		6
Golden Doodle		2
Golden Retriever		9
Golden Mix		1
Great Pyranese Mix	1	1
Hound Mix	17	
Husky Mix	1	
Labrador retriever		10
Lab Mix	1	7
Labradoodle		2
Lhasa Apso		1
Maltese		1
Maltese Mix		1
Miniature Poodle		1
Miniature Poodle Mix	2	
Miniature Schnauzer		5
Schnauzer Mix		1
Pitbull		6
Pitbull Mix	1	
Pointer Mix	4	
Pomeranian		1
Retriever Mix	51	
Rhodesian Ridgeback Mix	1	
Rottweiler mix	3	
German Shepherd Mix	24	
Shetland Sheepdog		2
Shih tzu		2
ShihTzu Mix	1	
Spaniel Mix	3	
Terrier Mix	74	3
West Highland Terrier		2
Wheaton Terrier		1
Yorkshire Terrier		2
Unknown	3	3
Total	260	101

### Sensor

**Fig. 1:**
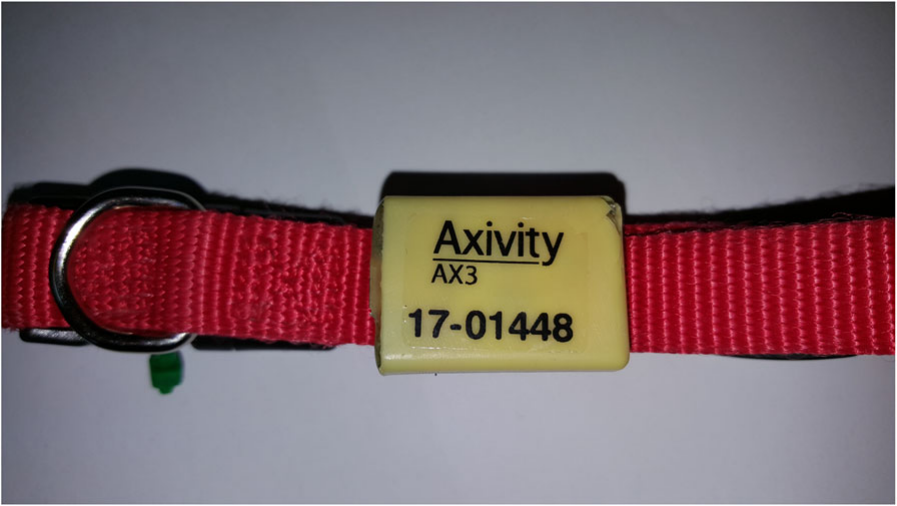
Axivity Ax3 Sensor

**Fig. 2:**
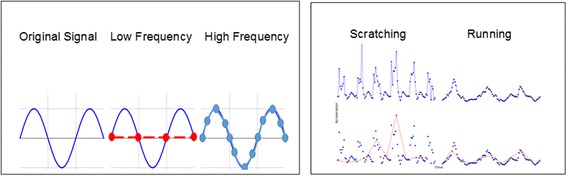
The importance of high frequency data sampling. Schematic of data sampled at low frequency misrepresents the original sinusoidal curve (left). Conversely, high frequency sampling enables an accurate digital representation of the original signal – facilitating capture of the necessary data fingerprint. Applied to actual data for dog behaviors scratching and running (right), data points sampled at high frequency are shown in blue, while the subset that would represent low frequency sampling is shown in red. The upper line graphs in blue connect the high frequency samples, and show a significant amount of detail from the captured signal. The lower line graphs connect the low frequency samples in red, and show far less detail making scratching and running examples not easily distinguishable. The low frequency sampling of the behaviors does not enable the accurate digital recreation of the signal, due to the loss of the high frequency components

**Fig. 3:**
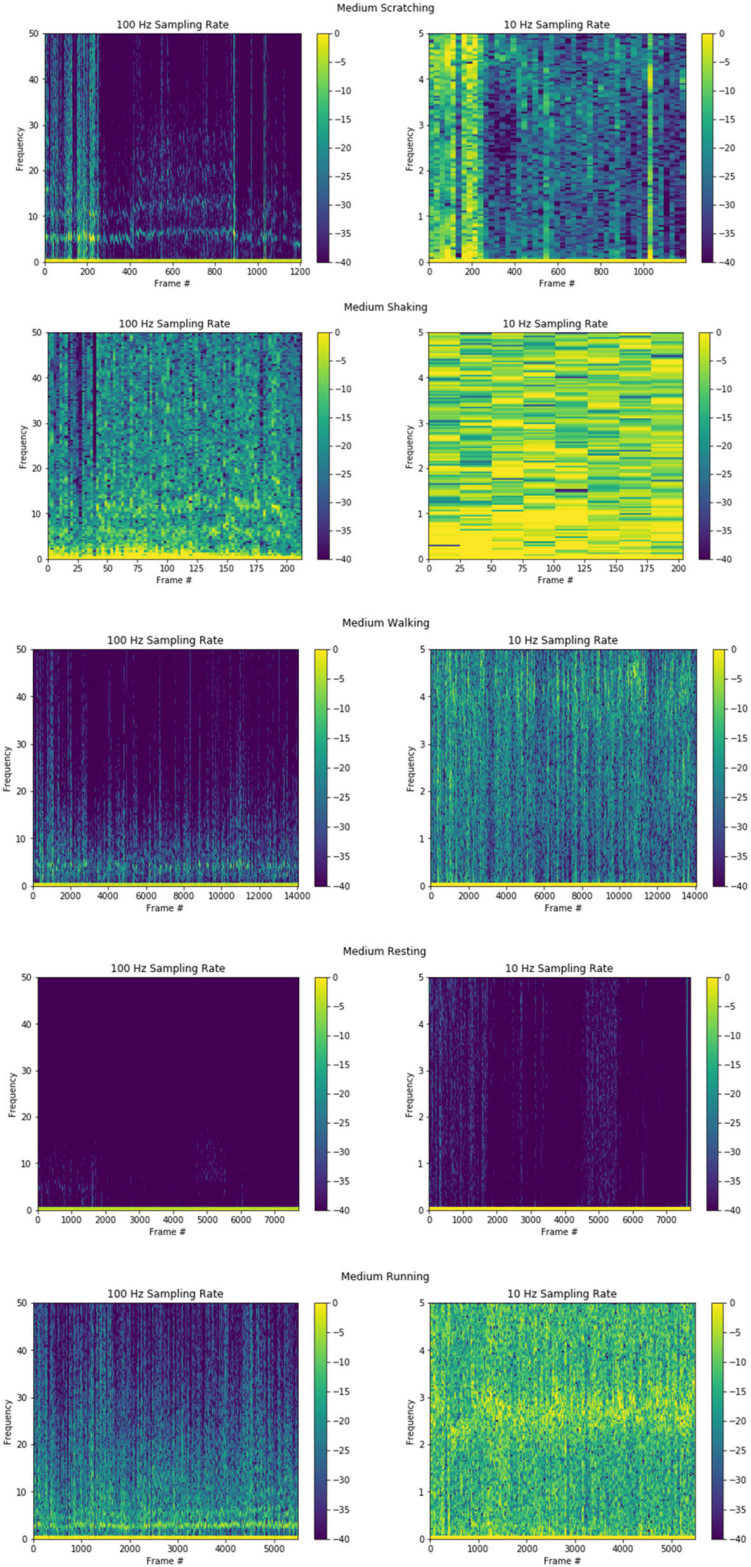
Spectrograms for behavior data. For each example, the amount of detail in the data is substantially less at low frequency than higher frequency. The higher amount of data and detail allows show greater differences between behaviors illustrating that higher frequency data sampling allows better differentiation and recognition of each individual behavior

The wearable sensor used for data collection was an AX3 data logger (Axivity Ltd, United Kingdom, Fig. 1). The sensor includes a micro-electro-mechanical systems (MEMS) 3-axis accelerometer and Flash based on-board memory. The on-board memory is capable of collecting and storing high density data (up to 100 Hz) for 14 days that was later offloaded via the sensor’s micro-USB port interface. The AX3 sensor was selected due to its ability to support configurable resolution/frequency data collection and to collect multidimensional data. This allowed the sensor to be set at sampling rates as low as 10 Hz (10 samples per second) up to 100 Hz. As illustrated in Fig. 2, the ability to collect data at a higher frequency allows more data points to be collected from a single event, presenting a more accurate representation of the original signal. Further analysis of spectrograms of behaviors collected, showed a significant difference in the high frequency content over lower frequency data that would be useful for distinguishing between behaviors (Fig. 3). The AX3 data logger was therefore configured at 100 Hz data sampling rate for data collection and computer algorithm development.

### Multidimensional sampling

**Fig. 4:**
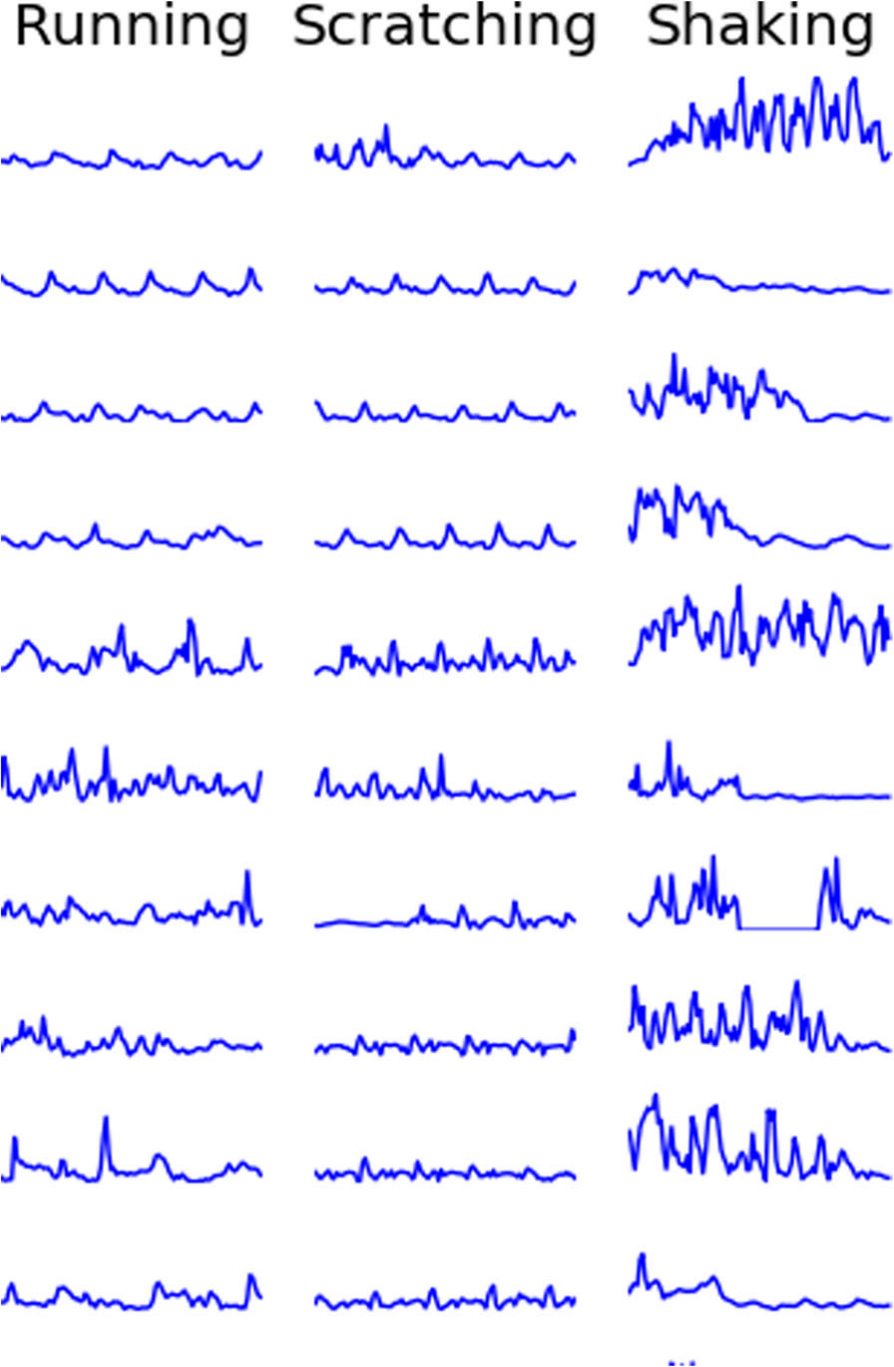
- Single-dimensional Data (1 s frames). Energy expended for each behavior is apparent but differences are difficult to distinguish from one another

**Fig. 5:**
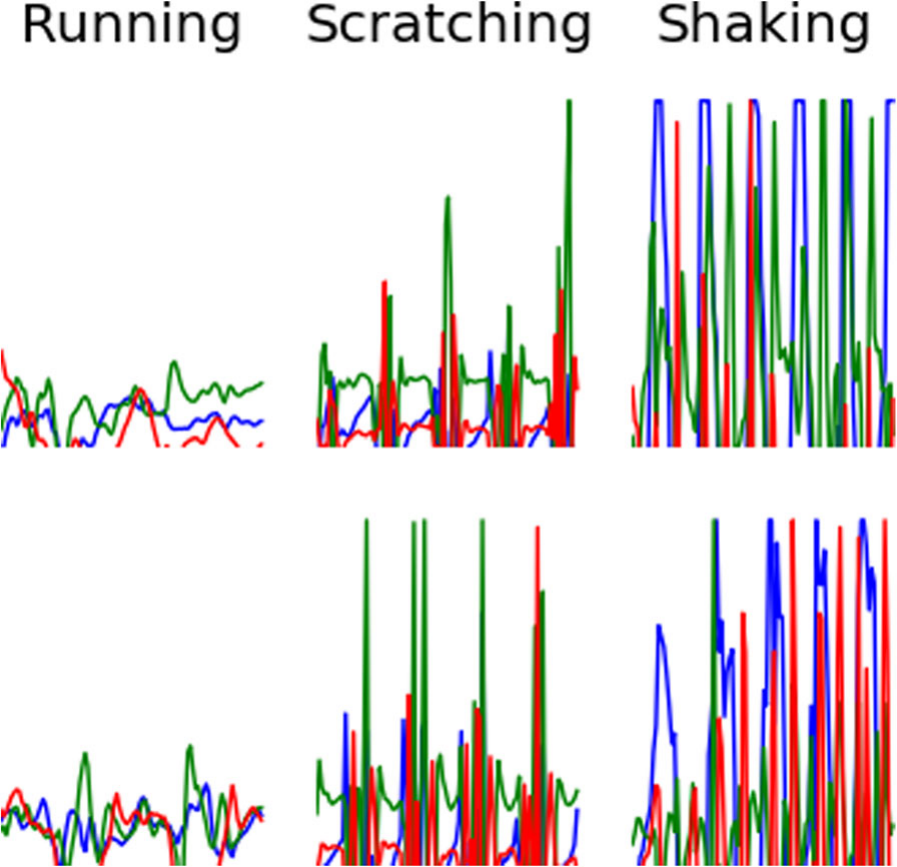
Multi-dimensional Data (1 s frames). Three dimensional data, demonstrates more obvious differences between behaviors

Sensor data captured can be represented as either single-dimensional data - measuring overall activity (Fig. 4) - or multi-dimensional data, evaluating data from x, y, and z axes (Fig. 5). With multidimensional sampling such as that used here, behaviors like running and scratching become much more differentiated and can be more easily identified as distinct behaviors.

Video recording was performed using a Nexus 7 tablet, Cannon VIXIA HF R600, and GoPro Hero4. Video capture devices were carried by the observer during data collection at HS1 and HS2 and tripod mounted on counter tops with view of the entire exam room at DR1. Sensors were attached to standard 1 in. collars prior to being applied to the dog. To synchronize sensor data and video documentation, an intentional 5 times shaking of the collar-attached sensor was performed within the video field of view when a data collection session was started and prior to the collar being applied to the dog. The collar was then applied to the dog and tightened as needed to provide a space equivalent to two finger-widths between the collar and the dog’s neck. Collars were rotated to position the sensor at the ventral cervical midline. Video recording continued during the data collection process and until the conclusion of the session. To conclude the recording session, the collar was removed from the dog, and 5 times shaking of the sensor was again performed within the video field of view. Duration of the recording session varied from 10 to 15 min at HS1 and HS2 and 15 to 60 min at DR1. Behaviors observed during the recording sessions included walking, running, resting (sitting, standing), eating, drinking, barking, chewing, urinating, digging, excreting (defecating), head shaking and scratching with a preponderance of normal behaviors at HS1 and HS2 and greater incidence of scratching and head shaking at DR1.

**Fig. 6:**
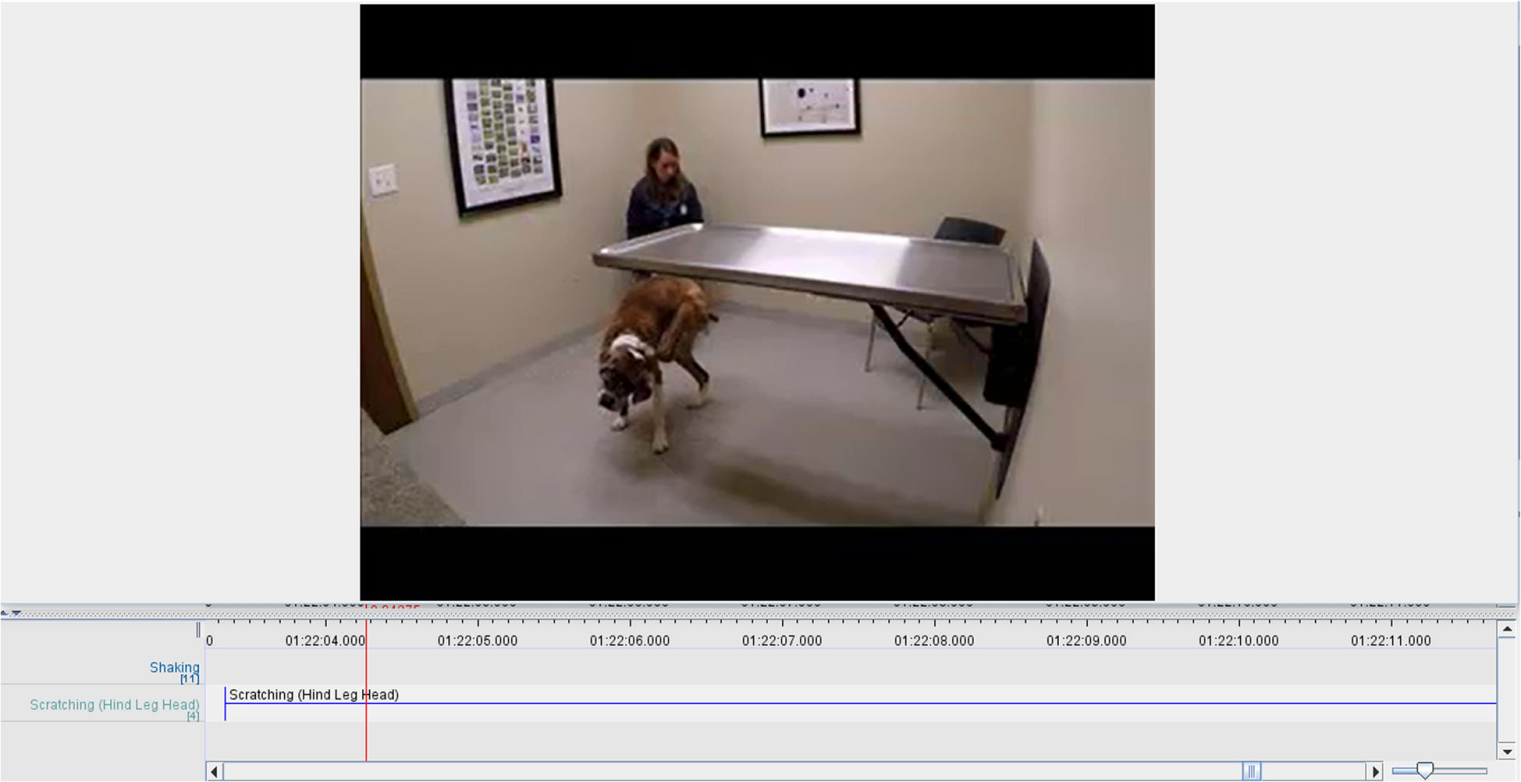
Video Annotation using ELAN® Linguistic Annotator (The Language Archive: The Netherlands) showing annotation of actions observed on video at exact start and stop times

**Fig. 7:**
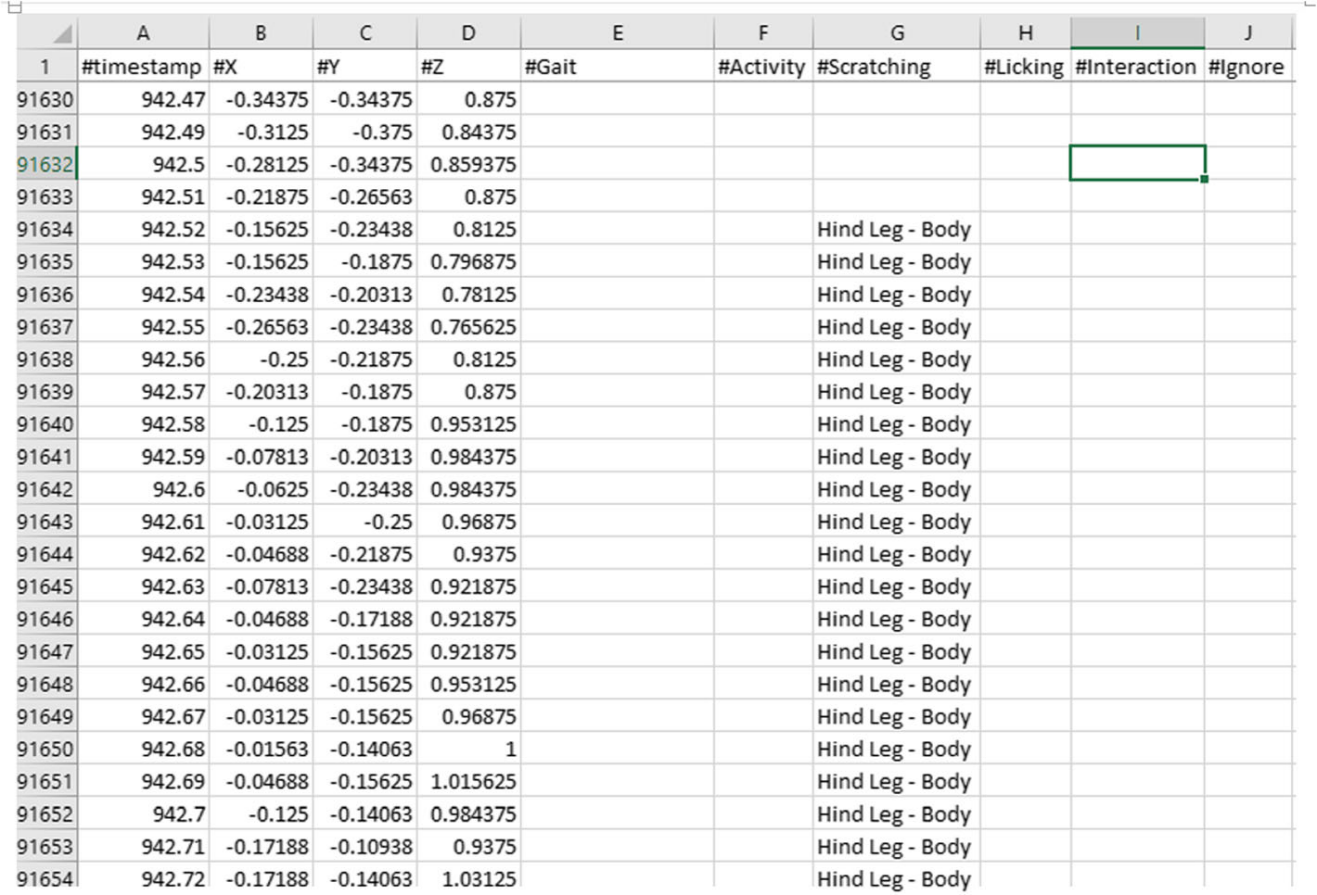
Data export from ELAN® with timestamp, x,y,z axis data and annotation labels

Each video collection segment was imported into ELAN Linguistic Annotator [7] and was manually annotated by two observers using a controlled vocabulary (Table 2) while blinded to sensor data. The common annotations from the two observers were exported to a single file (Fig. 6). Sensor data was also imported into ELAN and synchronized with video for each data collection session. Once annotated, data from each dog’s recording session was exported into a separate data file containing columns for time, sensor data, and the annotated behavior (Fig. 7). All non-annotated rows were dropped from each file, and then each data file was broken into one second frames of data, where the label of each frame was taken from the annotation. Each frame contained 100 records, each record representing 0.01 s of X, Y and Z accelerometer axis measurements. As data was aggregated, each frame was also labeled with its file of origin. This allowed data from a single dog’s collection event to be used only once in an algorithm’s training, testing, or validation set.

**Table 2:** Controlled Vocabulary for Video Editing

Gait	Activity	Scratching	Licking	Interaction	Ignore
Walking – Off Leash	Shaking	Front Leg – Body	Licking Paws	Petting	Not In View
Walking – On Leash	Drinking	Front Leg – Head	Licking Body		
Running – Off Leash	Eating	Hind Leg – Body	Other		Other
Running – On Leash	Chewing	Hind Leg – Head			
Laying – Resting	Barking	Other			
Sitting – Resting	Excreting				
Standing – Resting	Urinating				
	Digging

Prior to separating and cross-folding the data cohort, the population contained more than 110-thousand labeled one-second frames of activities (more than 30 h of annotated examples). To date, there is no data set of this magnitude in the animal health industry that has annotated behaviors.

### Algorithm development

Algorithms to identify behaviors were created using the Evolutionary Multi-objective Algorithm Design Engine (EMADE) framework, developed at Georgia Institute of Technology [8]. EMADE processes the data files through multiple generations of algorithm development cycles using a genetic programming approach. Genetic programming (GP) is a bio-inspired approach that allows computers to create a process or set of rules to be followed in calculations or problem solving (algorithms). It uses the concepts of survival of the fittest, mating, and mutation to create a population of candidate solutions. GP is distinguished from broader categories of genetic algorithms by its ability to change the structure of a program in addition to its parameters. To evaluate each candidate algorithm generated by EMADE, three criteria were chosen for simultaneous multi-objective optimization: false negative rate, false positive rate, and complexity of the algorithm. Because the first two are measures of error and our preference was something simpler over complex, the goal was to minimize all three of these objectives.

For the evolutionary machine learning process, the data collection was organized into two groups. The first was the set of data used to train and score the models to select the best candidate. The second set of data was withheld until the final algorithms were chosen and was then used to validate the performance of the algorithms on data to which they had not been previously exposed.

**Fig. 8:**
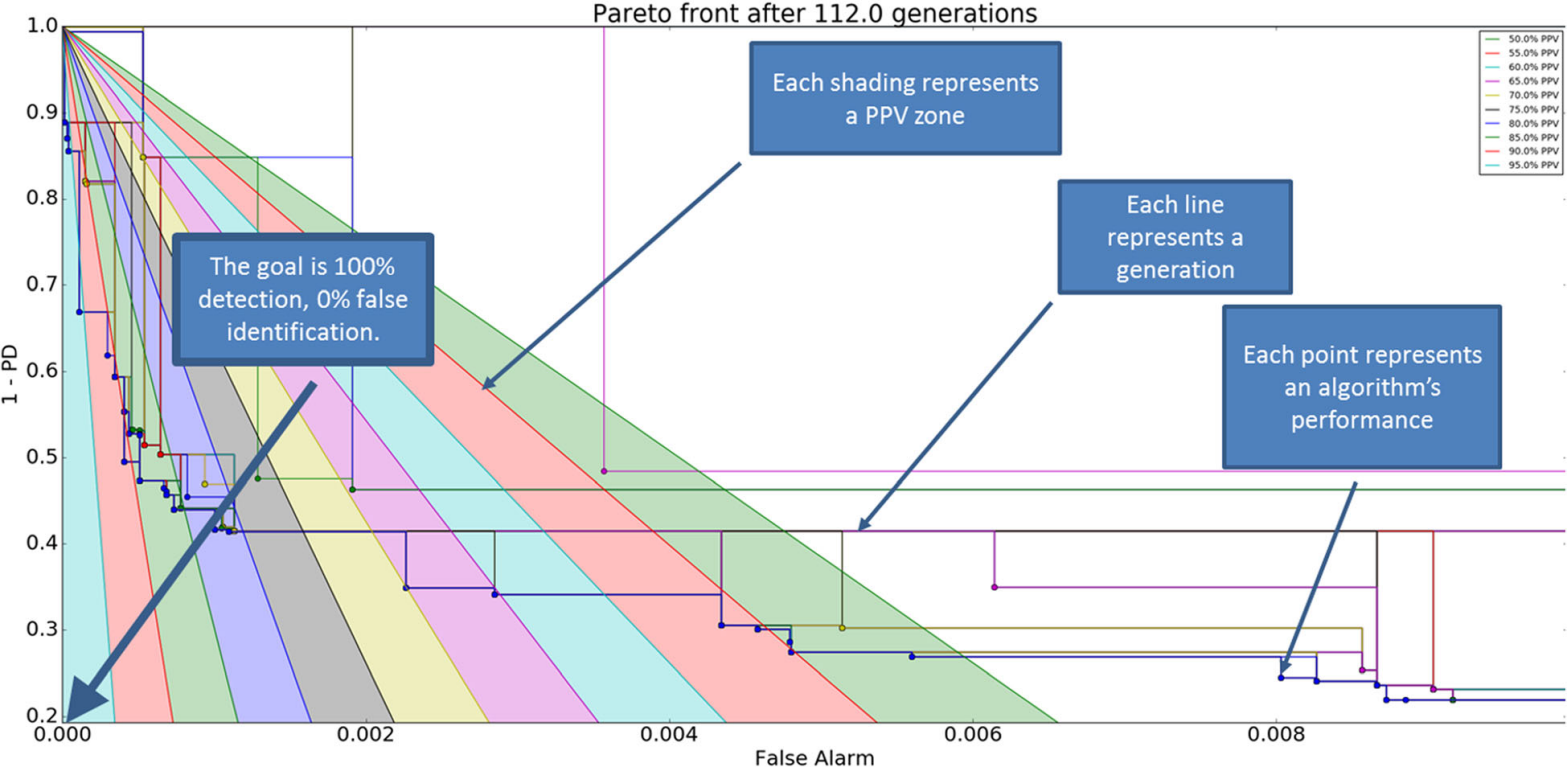
Pareto Front Graph for Head shaking illustrates successive generations of algorithms developed with a goal of maximum detection of events while minimizing false positives

A Pareto front graph for head shaking algorithm development (Fig. 8) displays sample algorithm performance associated with EMADE running through 112 generation cycles. The y-axis indicates the false negative rate (1 minus positive detection rate) of the behavior and the x-axis indicates the false positive rate of the behavior. The Pareto front graph illustrates that successive generation cycles result in new algorithm instances that progressively drive the next generation toward the lower left corner of the graph as it minimizes false negatives and false positives. Once the final algorithm was selected, new data was evaluated and scored to test the ability of the system to correctly identify behaviors.

Statistical analysis was performed and reported using the metrics of sensitivity (true positive rate), specificity (true negative rate), positive predictive value (PPV, precision), negative predictive value (NPV) and accuracy. The equations for each are shown below.

$$ {\displaystyle \begin{array}{l} Sensitivity=\frac{\# True\kern0.5em Positives}{\#\kern0.5em True\kern0.5em Positives\kern0.5em +\kern0.5em \# False\kern0.5em Negatives}\\ {} Specificity=\frac{\# True\kern0.5em Negatives}{\# True\kern0.5em Negatives\kern0.5em +\kern0.5em \# False\kern0.5em Positives}\\ {} PPV=\frac{\# True\kern0.5em Positives\kern0.5em }{\# True\kern0.5em Positives\kern0.5em +\kern0.5em \# False\kern0.5em Positives}\\ {} NPV=\frac{\# True\kern0.5em Negatives}{\# True\kern0.5em Negatives\kern0.5em +\kern0.5em \# False\kern0.5em Negatives}\\ {} Accuracy=\frac{\# True\kern0.5em Negatives\kern0.5em +\kern0.5em \# True\kern0.5em Positives}{\#\kern0.5em All\kern0.5em Negatives+\kern0.5em \# All\kern0.5em Positives}\end{array}} $$

## Results

### Animals

Data was collected from 361 dogs - 177 from HS1, 83 from HS2 and 101 from DR1. Because some dogs at HS1 and HS2 were present at more than one visit to the facility, video recordings of a previously recorded dog occurred on a repeat visit for some, resulting in a total of 472 recordings from these facilities and 573 data collection sessions overall. Exact weights were recorded for 148 of 260 dogs at HS1, HS2 and ranged from 9.5 to 88 lbs (mean 48.1, median 46lbs). Weights were recorded for 99 of 101 dogs from DR1 and ranged from 6.4 to 100.5 (mean 45.2, median 43.3 lbs). After video annotation, data labeling and frame aggregation, 112,082 frames of data were used for algorithm development and testing (Table 3). Of these, 2381 were identified as scratching frames and 896 as head shaking. These represented 2.1% and 0.80% of all frames respectively. Other behaviors identified included walking, running, laying, sitting, standing, drinking, eating, chewing, barking, excreting (defecating), urinating, digging, paw licking, body licking and petting. Algorithms were developed to best identify scratching and head shaking as described.

**Table 3:** Annotated data collection in labeled one second frames

Behavior	Total
Walking - Off Leash	45,118
Walking - On Leash	4230
Running - Off Leash	12,461
Running - On Leash	128
Laying - Resting	19,896
Sitting - Resting	4851
Standing - Resting	10,488
Head Shaking	897
Drinking	3257
Eating	2899
Chewing	33
Barking	212
Excreting	2404
Urinating	1187
Digging	12
Scratching	
Front Leg - Head	1
Hind Leg - Body	1246
Hind Leg - Head	1025
Other Scratching	109
Licking Paws	60
Licking Body	0
Petting	1568
Total	112,082

### Head shaking algorithm

After applying the testing data set, the head shaking algorithm selected for implementation showed sensitivity of 72.16% and specificity of 99.78%. In our data collection shaking made up 0.80% of the data, and this results in an overall accuracy of 99.56%, a positive predictive value of 72.57%, and a negative predictive value of 99.78%.

#### Scratching algorithm

The final scratching algorithm selected for implementation was validated to show sensitivity rate of 76.85% and specificity rate of 99.73%. In our data collection, scratching made up 2.12% of the data, resulting in an overall accuracy of 99.24%, a positive predictive value of 86.07%, and a negative predictive value of 99.50%.

## Discussion

The study reported here validated the use of an objective measurement tool for pruritus using a system that combines a wearable high frequency data collection sensor with newly developed computer algorithms designed to recognize specific behaviors. Previous studies using wearable sensors have been limited to differences in overall patient energy expenditures generated during activity [3–5]. As such, while energy expenditures may have been greater for pruritic atopic dogs, specific behaviors were neither identifiable nor attempted to be recorded. A recent attempt to identify specific behaviors in dogs using a small number of dogs (*n* = 13) did not specify accuracy for individual behaviors making it difficult to understand the positive predictive value of results [6].

A critical part of the correct identification of behaviors is the development of an algorithm classifier capable of identifying actions from the data collected. The ability of an algorithm classifier to properly identify behaviors is dependent on the ability of the development process to successfully identify a unique data “fingerprint” for each behavior. In developing these data fingerprints, the importance of the frequency at which data was collected became readily apparent. Sensor data sampling at too low of a frequency resulted in an inability to recreate the original signal and significant loss of detail. In contrast, high frequency sampling allowed the original signal to be readily observed and re-created without the loss of information. Similarly, multidimensional sampling provided more unique and distinguishable data to allow differentiation between behaviors. The more detailed data captured by high frequency and multidimensional sensor data collection are important drivers of performance in developing an algorithm classifier, and become the data input for algorithm development.

This highly detailed data, coupled with a genetic programming model of algorithm development allowed data generated by the sensor to be interpreted and correctly document behaviors with a high degree of accuracy.

In the study reported here, using a multidimensional high frequency sensing platform identified greater amounts of information about each activity but also provided much more detailed information for development of individual data fingerprints for each behavior. With more detailed data, a more accurate computer algorithm was developed to correctly identify the specific behaviors. Using this model, the identification and tracking of additional behaviors such as seizure or syncope activity should also be feasible, provided they can be documented in a significantly large population of dogs.

Pruritus is a complex sensation with a large variety of potential causes and ways it may be expressed. Scratching in dogs often occurs by the dog using a rear leg to scratch somewhere on its body. This behavior is a high energy repetitive action making it recognizable both to observers and the activity detected in the sensing platform. Similarly head shaking is also a high energy repetitive action that can be more readily detected. The extremely high specificity reported here (99%) for both of these behaviors documented that scratching or shaking, when noted by the sensor were very likely to have occurred. With a sensitivity of 76.85% and 82.16% for scratching and shaking, respectively, it is possible that that system may miss individual episodes of these behaviors. However, compared to current methods of owner assessment using the PVAS or other scale, or summaries of overall activity suspected to represent pruritus, this method far exceeds any other tool and is in fact an objective measurement. Ideally, this system should be implemented and continue to monitor an individual dog for weeks to months or longer. This would provide an individual baseline applicable to each dog allowing clinicians and researchers to identify differences noted during pruritic episodes and both before and after a particular treatment intervention.

Limitations of this study include the use of only scratching and head shaking as indicators of pruritus. Considering pruritus is often made up of multiple different behaviors, the addition of paw licking and chewing at a variety of sites would be valuable additions to the system. With the prevalence of paw licking especially in dogs with atopic dermatitis and canine adverse food reaction, however, these would provide a more comprehensive evaluation of pruritus as a whole and should be a focus of subsequent studies. Although owner assessments are highly variable and subjective, further research and clinical use are also warranted to correlate owner assessments of pruritus with sensor documentation.

Future research is needed to continue to develop this model to identify additional behaviors of both clinical and research importance. These may include seizures, drinking, urination and others. By being able to identify these behaviors, more objective data on frequency of these behaviors and subsequently their response to therapies could substantially aide the veterinary professional in patient management.

## Conclusions

Wearable sensors and the machine learning process are an exciting new frontier that offers tremendous benefits in veterinary medicine. With advances in these fields a variety of more objective real-time information can be developed to provide more accurate and timely medical assessments and decisions. Since pruritus has a significant effect on quality of life for pets and their owners, this information has potential to allow veterinarians to identify pruritic episodes quickly, suggest therapies and examinations to owners when needed and assess progress of medications and treatment plans. It also would allow the veterinary team to illustrate benefits of these treatments to pet owners during veterinary visits.

## Abbreviations

DR1: Animal Dermatology Clinic, 1453 Terrell Mill Rd SE, Marietta, GA 30067
HS1: Atlanta Humane Society, Howell Mill Campus, 981 Howell Mill Rd NW, Atlanta, GA 30318
HS2: Atlanta Humane Society, Mansell Campus, 1565 Mansell Rd, Alpharetta, GA 30009
